# Effects of metamizole, MAA, and paracetamol on proliferation, apoptosis, and necrosis in the pancreatic cancer cell lines PaTu 8988 t and Panc-1

**DOI:** 10.1186/s40360-017-0185-y

**Published:** 2017-12-06

**Authors:** Manuela Malsy, Bernhard Graf, Anika Bundscherer

**Affiliations:** Department of Anesthesiology, University Medical Center Regensburg, Franz Josef Strauss Allee 11, 93053 Regensburg, Germany

**Keywords:** Metamizole, Dipyrone, MAA, Paracetamol, Acetaminophen, Pancreatic carcinoma, Cancer, Proliferation, Apoptosis, Necrosis

## Abstract

**Background:**

Adenocarcinoma of the pancreas is one of the most aggressive cancer diseases affecting the human body. Recent research has shown the importance of the perioperative phase in disease progression. Particularly during this vulnerable phase, substances such as metamizole and paracetamol are given as general anesthetics and postoperative analgesics. Therefore, the effects of metamizole and paracetamol on tumor progression should be investigated in more detail because the extent to which these substances influence the carcinogenesis of pancreatic carcinoma is still unclear. This study analyzed the influence of metamizole and its active metabolites MAA (4-N-methyl-aminoantipyrine) and paracetamol on the proliferation, apoptosis, and necrosis of the pancreatic cancer cell lines PaTu 8988t and Panc-1 in vitro.

**Methods:**

Cell proliferation was measured by means of the ELISA BrdU assay and the rate of apoptosis by flow cytometry using the Annexin V assay.

**Results:**

Metamizole and paracetamol significantly inhibited cell proliferation in pancreatic cancer cells. After the addition of metamizole to PaTu 8988t cells, the rate of apoptosis was reduced after 3 h of incubation but significantly increased after 9 h of incubation.

**Conclusion:**

The oncogenic potential of pancreatic adenocarcinoma is mainly characterized by its extreme growth rate. Non-opioid analgesics such as metamizole and paracetamol are given as general anesthetics and postoperative analgesics. The combination of metamizole or paracetamol with cytotoxic therapeutic approaches may achieve synergistic effects. Further studies are necessary to identify the underlying mechanisms so that new therapeutic options may be developed for the treatment of this aggressive tumor.

## Background

Adenocarcinoma of the pancreas is one of the deadliest cancers worldwide with an overall life expectancy of 6 months [1]. Over the past few years, important advances have been made in the molecular and biological understanding of pancreatic carcinoma [2]. Unfortunately, however, the clinical outcome has not significantly changed for patients with pancreatic carcinoma [3]. The main reasons for the poor prognosis of pancreatic carcinoma are early metastasis, so far insufficient diagnostic and therapeutic options, as well as a high recurrence rate [4]. Pancreatic carcinoma is also known for its extremely rapid growth [5]. A further therapeutic option apart from chemotherapy or radiation treatment is surgical removal of the tumor. However, investigations over the past few years have shown that the perioperative period is a particularly vulnerable phase marked by facilitation of tumor progression and metastasis [6]. The combination of surgical intervention, a perioperatively compromised immune system, and drug therapy increases the risk of tumor dissemination, thus exerting a negative impact on disease progression in oncological patients [7, 8]. Precisely at this vulnerable stage, substances such as metamizole and paracetamol are administered as anesthetics or postoperative analgesics [9, 10]. However, the direct effects of these substances on the tumor progression and carcinogenesis of pancreatic carcinoma are still unclear and require further investigations.

Metamizole (dipyrone) is a pyrazol derivative that belongs to the group of non-acidic, non-opioid analgesics [11]. In the organism, separation of the sulfonate group and the associated methylene group activates the actually effective substance 4-methyl-aminoantipyrine (MAA). Metamizole is the preferred first-line non-opioid analgesic in many parts of the world, such as most EU countries and Latin American countries. However, other countries such as the United States, the United Kingdom, Sweden, and most recently India have banned metamizole because of its side effects (amongst others, agranulocytosis, leukopenia, and deterioration in renal function) [12, 13]. In these countries, patients are preferably given paracetamol (acetaminophen), an aminophenol derivative.

How exactly metamizole or paracetamol work in the organism remains unclear. The substances are known to act as a cyclooxygenase-2 inhibitor [14, 15]. Cyclooxygenases catalyze the conversion of arachidonic acid to endoperoxide, the pre-stages of prostaglandin, thromboxane A2, and prostacyclin [16]. Another possibility discussed by scientists is involvement of the 5-HT or opioid metabolism, the cGMP signal pathway, or blockade of TRPA1 ion channels [17–19]. The extent to which metamizole or paracetamol influence the carcinogenesis of pancreatic carcinoma is so far unclear.

Aim of this study was to analyze the influence of metamizole with its active metabolites MAA (4-N-methyl-aminoantipyrine) and paracetamol on the proliferation, apoptosis, and necrosis of pancreatic cancer cell lines PaTu 8988t and Panc-1 in vitro.

## Methods

### Cell lines

The human pancreatic cancer cell lines PaTu 8988t and Panc-1 were obtained from Professor Ellenrieder (Philipps University of Marburg, Germany). The pancreatic cell line PaTu 8988t was established from a liver metastasis of a primary pancreatic adenocarcinoma and grown in structural characteristics of highly differentiated primary pancreatic adenocarcinoma. In contrast, the human cell line Panc-1 was obtained from a pancreatic carcinoma of ductal origin exhibiting a low level of differentiation.

PaTu 8988t and Panc-1 cells were maintained in Dulbecco’s modified Eagle’s medium (Sigma-Aldrich), which was supplemented with 10% fetal calf serum (FCS) (Sigma-Aldrich) and 5% Myco Zap (Lonza Verviers SPRL). Cells were cultured in humidified CO_2_ atmosphere (5%) at 37 °C and maintained in monolayer culture. Experiments were done with cells at ~65–75% confluence.

### Reagents

Metamizole was purchased from Fluka, MAA from Sigma-Aldrich, and paracetamol from Merck. Final concentrations were obtained by diluting drugs in standard growth media. All solutions were prepared freshly prior to use.

### Cell proliferation

For cell proliferation analysis the cell proliferation ELISA BrdU (Roche applied science) was applied. In brief, cells were incubated with 100 μL of the test compounds for 48 h (1–500 μM of metamizole, 1–500 μM of MAA, 1–1000 μM of paracetamol, or 250 μM of metamizole, and 250 μM of paracetamol). 5 mM ASS was used for positive control and standard culture medium was used for negative control. After 32 h of incubation, cells were additionally treated with BrdU labeling solution for the last 16 h. After fixing the cells and denaturating DNA, cells were incubated with Anti-BrdU-POD solution for 90 min. The antibody conjugates were removed in three washing cycles. Immune complexes were detected by means of TMB substrate for 15 min and quantified by measuring absorbance at 405 nm and 490 nm. All tests were performed in duplicates with 8 wells per treatment group and repeated three times.

### Apoptosis analysis

Apoptosis assays with Annexin V staining were conducted according to the manufacturer’s instructions (BD Pharming). In brief, PaTu 8988t and Panc-1 cells were incubated with 250 μM of metamizole, MAA, paracetamol or metamizole, and paracetamol. Standard growth medium was used for negative control. After 0 h, 3 h, 6 h, 9 h, 12 h, 16 h, 24 h, or 48 h incubation time, floating cells were preserved by decanting supernatant. Adherent cells were rinsed with warm PBS (Sigma-Aldrich) and detached by standard trypsinization. Afterwards, harvested and floating cells were mixed, washed, and resuspended in binding buffer at a final concentration of 10^5^ cells/ml. 5 μL of FITC Annexin and 5 μL of propidium iodide were added to 100 μL of the cell suspension containing 10^5^ cells, followed by 15 min incubation at room temperature protected from light. 400 μL of binding buffer were added and cells were analyzed by flow cytometry using FACS Calibur (BD Bioscience) and Cellquest Pro software (BD Bioscience). All tests were performed in duplicates and repeated three times. 1 μM of staurosporine, an often employed method for inducing apoptosis, was used as a positive control for the testing procedure and induced significant apoptosis in the pancreatic cancer cells.

### Statistical analysis

Results are expressed as mean ± SD. The non-parametric Mann Whitney U-test was used for statistical evaluation of the data. Differences were considered statistically significant at *p* values of <0.05. IBM SPSS Statistics (Vs. 23; IBM New York, US) and Excel Vs. 2013 (Microsoft, Redmond, USA) packages were employed for statistical analysis.

## Results

### Cell proliferation behavior

The pancreatic cancer cell lines PaTu 8988t and Panc-1 were either stimulated with 1–500 μM of metamizole (a), 1–500 μM of MAA (b), 1–1000 μM of paracetamol (c), or 250 of μM metamizole, and 250 μM of paracetamol (d) for 48 h.

**Fig. 1:**
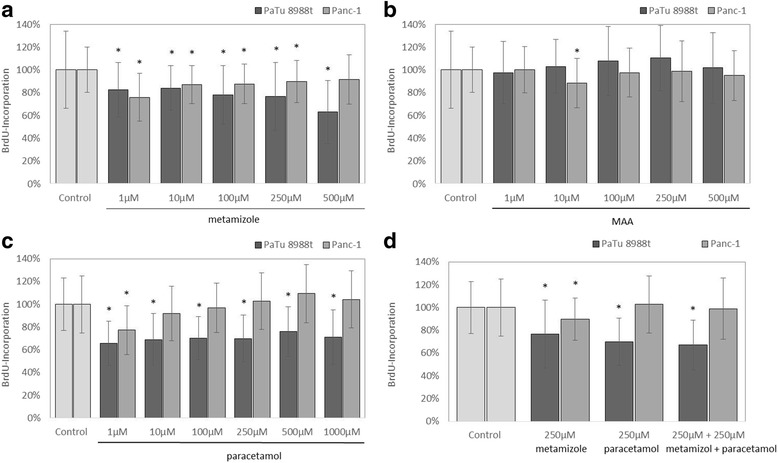
Effect of metamizole (**a**), MAA (**b**), paracetamol (**c**), and the combination of metamizole and paracetamol (**d**) on the proliferation of the pancreatic cancer cell lines PaTu 8988t and Panc-1 after 48 h incubation. The proliferation rate was determined by means of proliferation BrdU assays. 100% correspond to untreated control. (*) *p* < 0.05 in comparison to untreated control

As a result, proliferation was significantly inhibited in the PaTu 8988t cell line after incubation with 1–500 μM of metamizole **(**Fig. 1a **)** and 1–1000 μM of paracetamol **(**Fig. 1c). In PaTu 8988t cells, the combination of 250 μM of metamizole and 250 μM of paracetamol also significantly reduced cell growth **(**Fig. 1d **)**.

In the pancreatic cancer cell line Panc-1, proliferation was significantly inhibited with concentrations of 1 μM, 10 μM, 100 μM, and 250 μM of metamizole **(**Fig. 1a). A further slight inhibition was achieved with 10 μM of MAA **(**Fig. 1b **)** and 1 μM of paracetamol in comparison to the untreated control group **(**Fig. 1c). No other significant changes in the proliferation rate were observed using the other concentration.

### Analysis of apoptosis and necrosis

**Fig 2:**
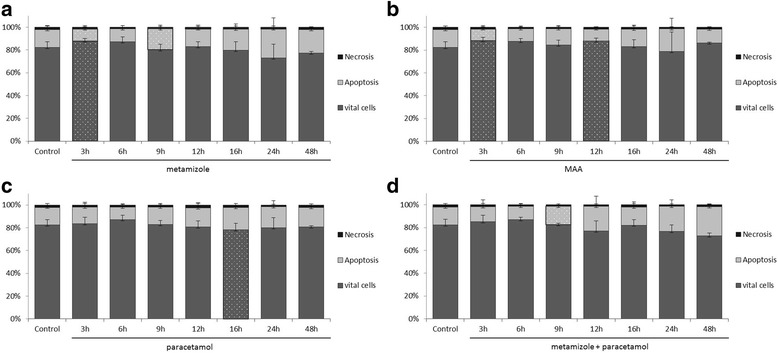
The effects of metamizole (**a**), MAA (**b**), paracetamol (**c**) and the combination of metamizole and paracetamol (**d**) on apoptosis in the pancreatic cancer cell lines PaTu 8988t (Fig. 2) and Panc-1 (Fig. 3) in vitro. For apoptosis analysis, cancer cells were stained with Annexin V. (*) indicates statistical significance at *p* < 0.05 compared to untreated control.

**Fig 3:**
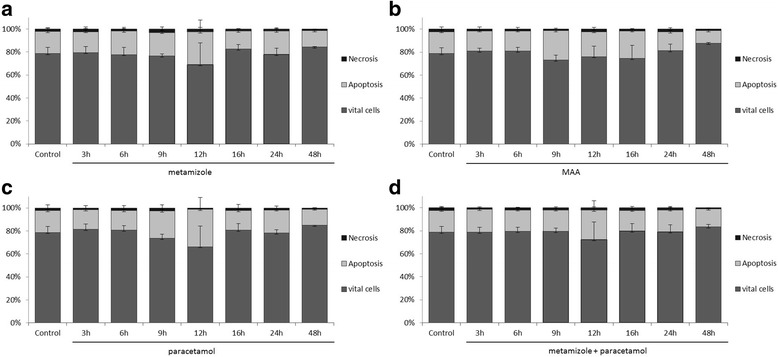
The effects of metamizole (**a**), MAA (**b**), paracetamol (**c**) and the combination of metamizole and paracetamol (**d**) on apoptosis in the pancreatic cancer cell lines PaTu 8988t (Fig. 2) and Panc-1 (Fig. 3) in vitro. For apoptosis analysis, cancer cells were stained with Annexin V. (*) indicates statistical significance at *p* < 0.05 compared to untreated control.

The Annexin V staining apoptosis assay was used to determine whether stimulation with metamizole, MAA, and paracetamol or the combination of metamizole and paracetamol caused apoptosis or necrosis in the pancreatic cancer cell lines PaTu 8988t **(**Fig. 2 **)** and Panc-1 **(**Fig. 3 **)**.

In the pancreatic cancer cell line PaTu 8988t, incubation with metamizole (a) and MAA (b) for 3 h **(**Fig. 2 **)** reduced the apoptotic cell fraction phase to 70% and 68% compared to untreated samples. In contrast, the apoptosis rate was significantly increased after 9 h of incubation with metamizole (a) and with the combination of metamizole and paracetamol (d).

The other incubation times with metamizole and MAA did not yield any changes in the apoptosis rate, as well in Panc-1 pancreatic cancer cells. Paracetamol by itself did neither influence apoptosis nor necrosis in pancreatic tumor cells.

The positive control staurosporine induced apoptosis in both cell lines in a time-dependent manner (Figure not shown).

## Discussion

The fact that many substances without any primary indication for treating tumor diseases show anti-tumoral behavior has been known for several years. Next to direct effects on tumor cells, such as inhibition of cell proliferation or induction of apoptosis, clinical studies have also shown the modulation of peri-tumoral stroma [20]. Interactions between the tumor and the surrounding stroma play a vital role in tumor progression. Changes in the surrounding tissue provide ideal conditions for tumor growth, invasion, and subsequent metastasis [21]. Furthermore, inflammatory processes correlate with the development of precancerous lesions [22]. The fact that the presence of inflammation facilitates the induction of carcinogenesis has been described in many literature reports [23]. A further independent risk factor for the development of pancreatic carcinoma is chronic pancreatitis [24].

Therefore, preventive effects have been expected from non-steroid anti-inflammatory drugs (NSAIDs) because of their anti-oxidative and anti-inflammatory properties [25]. NSAIDs primarily inhibit activity of cyclooxygenase (COX), thus influencing the synthesis of prostaglandins as the central regulators of inflammation [26]. However, the risk of pancreatic carcinoma is neither reduced by the supplementation of antioxidants [27] nor by the administration of non-steroidal anti-rheumatic drugs [28, 29]. Thus, the preventive intake of such medications is not recommended in the current guidelines for the treatment of pancreatic carcinoma [10].

In 1999, Tucker et al. showed increased COX-2 levels in pancreatic carcinoma [30]. Similar increases were also found in other human tumor cell lines [31], so that the inhibition of cyclooxygenase 2 in the context of malignancies has become the focus of tumor research, which is shown by the number of publications in the literature [32–34]. Yip-Schneider et al. found that the selective COX-2 inhibitor celecoxib significantly inhibits cell proliferation and induces apoptosis in pancreatic tumor cells [35]. Li et al. showed that celecoxib inhibits proliferation, invasion, and migration in Panc-1 pancreatic tumor cell lines [36].

However, these results could not be confirmed in clinical studies. In a phase II study, Ferrari et al. reported that the combination of celecoxib and gemcitabine yielded good clinical benefits and stable disease [37]. In contrast, Dragovich et al. did not find any significant improvement in the survival time of patients with metastatic cancer [38]. Thromboembolism is also a common complication in patients with pancreatic carcinoma [39]. Intrinsic hypercoagulability and activating procoagulant factors, such as the tissue factor (TF), platelet factor 4 (PF4), and plasminogen activator inhibitor type 1 (PAI-1) often cause deep vein thrombosis, pulmonary embolism, disseminated intravascular coagulation, portal vein thrombosis, or arterial thromboembolism [40]. Scientists have discussed the activation of the coagulation systeme not only as a probable concomitant condition of the disease but as being directly related to facilitating tumor growth and angiogenesis [41]. Because of their side effects, COX-2 inhibitors may increase such adverse effects, thus promoting tumor progression.

Metamizole and paracetamol do not have any selective effect on cyclooxygenase and thus do not cause thromboembolism. On the contrary, these substances have often been reported to inhibit platelet aggregation [42, 43].

Therefore, it is all over more important to analyze the effect of these two substances in clinically relevant concentrations on the carcinogenesis of pancreatic carcinoma.

The pyrazolone derivate metamizole is rapidly hydrolysed to its active metabolite 4-methylaminoantipyrine (MAA). After oral administration of 1 g metamizole maximal plasma concentration of 17,3 +/− 7,5 mg/l was measured within 1–2 h. Peak plasma concentrations of MAA of 62,2 +/− 15,9 mg/l (≈210–350 μM) were obtained after intravenously injection of 1 g metamizole [44]. In a pharmacological study patients received 1 g acetaminophen every 6 h intravenously. Peak plasma concentrations after the first administration was 95 +/− 36 μM, after the 4th intravenously dose concentrations of 210 +/− 84 μM were measured [45].

In our study, cell proliferation in PaTu 8988t pancreatic tumor cell lines could be inhibited by administration of metamizole. No dose-dependent effect could be observed and even small doses seem to be sufficient. And also the combination of 250 μM of metamizole and 250 μM of paracetamol significantly reduced cell growth.

Interestingly, inhibition of proliferation was not possible with 4-methylaminoantipyrine (MAA), the actually active substance of metamizole. According to its mode of action, metamizole in the organism is rapidly hydrolyzed to MAA that is then acetylated to 4-formylaminoantipyrine (4-FAA), 4-aminoantipyrin (4-AA), or 4-acethylaminoantipyrine (4-AAA). Here, all metabolites are pharmacologically active [46], so that inhibition is possibly induced by 4-FAA, 4-AA, or 4-AAA. In 2013, Shao et al. reported on the anti-proliferative effects of metamizole in the cancer cell lines A549 and HeLa [47]. The anti-proliferative effects of aspirin, indometacin, parecoxib, and ibuprofen in animal models have also been described in the recent literature [48, 49]. However, no clinical studies are yet available on these substances as a support therapy in addition to chemotherapy.

In the pancreatic tumor cell lines PaTu 8988t and Panc-1, paracetamol has only a minor but still significant anti-proliferative effect. Striking is that the proliferation was significantly inhibited in the PaTu 8988t cell line after incubation with 1–1000 μM of paracetamol but in Panc-1 only an inhibition was achieved with 1 μM of paracetamol in comparison to the untreated control group. The difference of the two cell lines is the grade of differentiation. The pancreatic cell line PaTu 8988t was established from a liver metastasis of a primary pancreatic adenocarcinoma and grown in structural characteristics of highly differentiated primary pancreatic adenocarcinoma. In contrast, the human cell line Panc-1 was obtained from a pancreatic carcinoma of ductal origin exhibiting a low level of differentiation.

The effect of paracetamol on carcinogenesis is even more unanswered because of the limited data currently available in the literature. On the one hand, Tan et al. did not find any connection between paracetamol and the development of pancreatic carcinoma [50]. The increased expression of differentiation markers in breast cancer indicates that paracetamol changes tumor cells into a more benign type with less tumor growth, limited invasion capacity, and increased sensibility to anti-tumoral substances [51]. In contrast, therapeutic doses of paracetamol increase cell proliferation in lung cancer [52] and stimulate DNA synthesis in breast cells sensitive to estrogen [53]. Many more clinical studies are necessary to identify the effect of these two substances on carcinogenesis and to characterize their underlying mechanisms.

## Conclusion

The perioperative phase plays a vital role in the progression of tumor diseases due to the combination of perioperative immunosuppression, release of tumor cells by surgical manipulation, and increased concentrations of growth factors [6]. Particularly in this vulnerable phase, a multitude of substances is administered as anesthetics and postoperative analgesics, whose effects on tumor progression have to be thoroughly investigated. In the current study, the influence of metamizole, MAA, and paracetamol on cell proliferation, apoptosis, and necrosis could be shown in vitro in the pancreatic cancer cell lines PaTu 8988t and Panc-1. Therapeutic doses of metamizole and paracetamol inhibit proliferation in the pancreatic cancer cell line PaTu 8988t. A combination of metamizole or paracetamol with cytotoxic therapy may achieve synergistic effects. Further studies are necessary to identify the underlying mechanisms to be able to establish new therapeutic options for this aggressive type of tumor.

## Abbreviations

4-AA: 4-Aminoantipyrin
4-AAA: 4-Acethylaminoantipyrine
4-FAA: 4-Formylaminoantipyrine
COX-2: Cyclooxygenase-2
FCS: 10% fetal calf serum
MAA: 4-Methylaminoantipyrin
NSAIDs: Non-steroid anti-inflammatory drugs
PAI-1: Plasminogen activator inhibitor type 1 -
PF4: Platelet factor 4
TF: Tissue factor
